# Paravertebral Mass and Diffuse Lymphadenopathy in a Patient with Pyruvate Kinase Deficiency: Malignancy or Alternative Etiology?

**DOI:** 10.7759/cureus.4849

**Published:** 2019-06-06

**Authors:** Hira Shaikh, Veli Bakalov, Soorih Shaikh, Ali Amjad

**Affiliations:** 1 Internal Medicine, Allegheny Health Network, Pittsburgh, USA; 2 Oncology, Allegheny Health Network, Pittsburgh, USA

**Keywords:** pyruvate kinase deficiency, extramedullary hematopoiesis, paravertebral mass, infection, medistinal mass, intraabdominal lymphadenopathy

## Abstract

Extramedullary hematopoiesis is common in chronic hemolytic anemias such as pyruvate kinase deficiency. It is commonly associated with hepatosplenomegaly or lymphadenopathy; however, it can rarely also present as a mass in the chest, abdomen, or paraspinal region. Here, we present a case of an adult patient with pyruvate kinase deficiency and history of splenectomy. He presented with sepsis and brisk leukocytosis secondary to pneumonia and was also found to have diffuse intraabdominal lymphadenopathy along with a paravertebral mass. The radiological findings raised concerns for a systemic lymphoproliferative disorder and there was a suggestion for further workup with a biopsy. However, given the patient’s underlying pyruvate kinase deficiency, we hypothesized that the paravertebral mass is likely a result of extramedullary hematopoiesis in the setting of bone marrow stress from infection and ongoing hemolysis; thus, we decided against biopsy. Repeat imaging six weeks after the presentation showed resolution of the paravertebral mass, which consolidated our hypothesis. This highlights the importance of avoiding invasive diagnostic procedures in asymptomatic patients with chronic hemolysis who may present with diffuse mass lesions.

## Introduction

An incidental finding of a paravertebral mass and lymphadenopathy may raise concerns for lymphoproliferative conditions, cancers, infectious or inflammatory etiologies, and clinical correlation of the findings is crucial. Here, we present a case of the patient with pyruvate kinase deficiency who presented with sepsis secondary to pneumonia and was also found to have a paravertebral mass along with diffuse lymphadenopathy.

## Case presentation

We present a case of a 40-year-old male with a history of pyruvate kinase deficiency that was diagnosed at birth. He required multiple transfusions growing up as a child due to recurrent episodes of hemolysis that led to splenectomy at the age of 33 years. He had not required transfusions since then. Medications included folic acid, vitamin D, and aspirin. 

The patient presented to the emergency department with fevers accompanied by sore throat, pleuritic chest pain, and productive cough for four days. On presentation, he had a fever of 103˚ F and was hypotensive to 97/55 mmHg. Bibasilar rhonchi were evident on physical exam but there was no lymphadenopathy. Blood work was remarkable for white blood cells (WBC) of 35,000 cells/µl, hemoglobin of 11.9 g/dL, lactate dehydrogenase (LDH) elevated to 249 U/L and total bilirubin to 2.8 mg/dL. 

**Figure 1:**
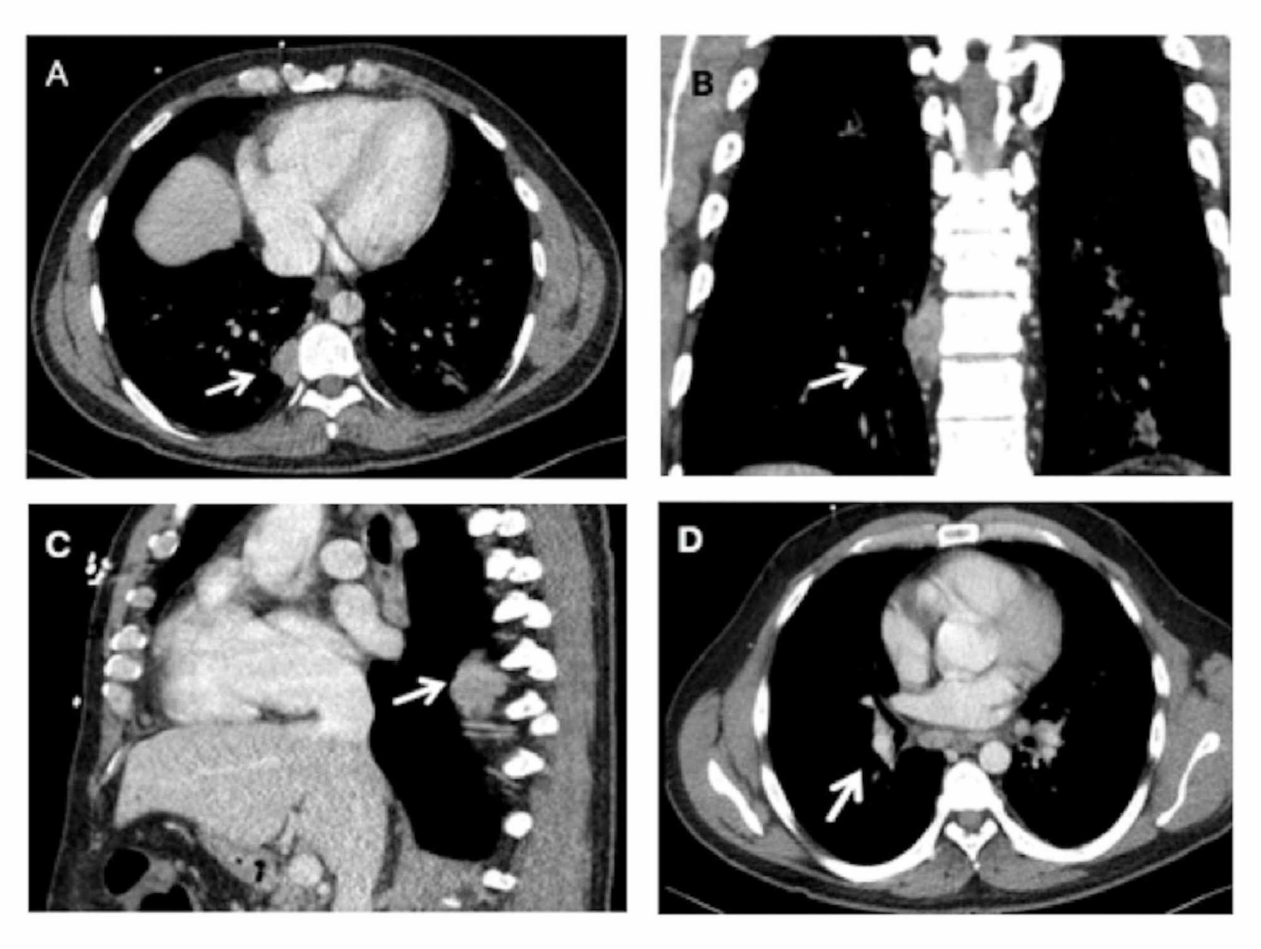
(A) Axial, (B) coronal, and (C) sagittal view of the right paravertebral posterior mediastinal mass or lymphadenopathy. (D) Diffuse mediastinal lymphadenopathy on the chest CT scan

Contrast-enhanced computerized tomography (CT) scans showed mild cervical lymphadenopathy, and bilateral multi-lobar ground glass opacities consistent with pneumonia. Of particular interest was a right-sided paravertebral mass near T9 measuring up to 3 x 2 cm, and mediastinal and mesenteric lymphadenopathy, largest measuring to 2 x 1 cm, as shown in Figure 1. Our differential diagnosis for the paravertebral mass included lymphoma, metastasis, neurogenic tumor, mesenchymal tumor, and abscess. Microbiological evaluation including blood and sputum culture resulted negative, so did the serologies for Epstein-Barr virus (EBV), cytomegalovirus (CMV), and human immunodeficiency virus (HIV) infection. Peripheral blood flow cytometry did not show monoclonal proliferation. 

The patient was started on broad-spectrum antibiotics, with IV vancomycin, and piperacillin and tazobactam. Fever and leukocytosis improved within 24-48 hours and he was subsequently discharged to home. Lymph node biopsy was deferred, with a plan to follow up outpatient with repeat imaging in 6-8 weeks to document the progression of lymphadenopathy after the resolution of the infection.

Repeat CT chest done within six weeks showed resolution of the paravertebral mass and mediastinal lymphadenopathy, and showed small residual pulmonary nodules. The patient continued to be asymptomatic.

## Discussion

Pyruvate kinase deficiency results in energy depletion in the red blood cells (RBCs) and subsequently causes chronic hemolytic anemia [1]. Extramedullary hematopoiesis frequently occurs as compensation for stressed bone marrow [2]. Extramedullary hematopoiesis is commonly associated with bone marrow failure syndromes like myelofibrosis but has also been reported in chronic hemolytic anemias such as thalassemia, sickle cell disease, and hereditary spherocytosis [3-5]. In the majority of adults, extramedullary hematopoiesis develops in the liver and spleen [6-7]. Non-hepatosplenic extramedullary hematopoiesis can present in lymph nodes and a wide variety of organs such as thyroid, heart, kidneys, central neural system, and others [8-11].

Our patient presented with brisk leukocytosis, diffuse lymphadenopathy, and a para-osseous mass in the setting of infection. The imaging findings were naturally concerning for lymphoproliferative disorder and there was a suggestion for further workup with a biopsy for tissue diagnosis. However, with the history of splenectomy and pyruvate kinase deficiency, we hypothesized that the masses represent areas of extramedullary hematopoiesis to compensate for the ongoing hemolysis in the setting of infection [12-13]. Therefore, we elected to defer tissue diagnosis with biopsy until after complete resolution of the infection. Follow up imaging showed resolution of these areas, thus consolidating our idea. 

In asymptomatic patients with paraspinal masses with high suspicion of extramedullary hematopoiesis, invasive diagnostic procedures frequently are not necessary and are associated with complications secondary to the high vascularity of these masses [4-5, 14-15]. Combining the clinical picture and laboratory studies with CT or MRI imaging is often highly accurate to establish the diagnosis of extramedullary hematopoiesis in patients with chronic hematologic conditions [14]. Although rarely symptomatic, it can develop into complications and be potentially fatal; for instance, rupture of nodules could result in hemothorax [7]. Routine follow-up and imaging are reinforced. On the other hand, surgery and radiation therapy has been recommended in symptomatic patients [16]. Surgery is preferred when decompression is required in those with paraspinal masses causing spinal cord compression and has an added benefit of achieving a histological diagnosis. However, it comes with the risk of major hemorrhage due to the high vascularity of the mass, and it has a higher incidence of recurrence. Also, total resection of the mass can lead to worsening of anemia since many with chronic hemolytic anemias depend on extramedullary sites to compensate for ongoing hemolysis. While radiation is less invasive, it has been known to cause a reduction in the bone marrow activity and confers the risk of secondary malignancies. Hydroxyurea is another option for the management of such masses [17]. Our patient was not symptomatic from the paravertebral mass or lymphadenopathy, and he improved with the treatment of pneumonia.

Usually, extramedullary hematopoiesis, if secondary to congenital hemolytic anemia, presents in childhood, but there have been rare cases [18], including ours, that present in adulthood. This makes the condition more challenging. Aggressive workup of these conditions has led to incidental findings in the past which are mostly clinically irrelevant and lead to patient anxiety; thus, there is a need keep a broad differential in such patients.

## Conclusions

Awareness of clinicians about the possibility of extramedullary hematopoiesis in patients with chronic hematologic conditions such as pyruvate kinase deficiency is important. Invasive procedures or aggressive therapeutic approaches are usually unnecessary and should be avoided in asymptomatic patients, and close follow-up should be encouraged. 
